# A Hypothesis-Testing Framework for Studies Investigating Ontogenetic Niche Shifts Using Stable Isotope Ratios

**DOI:** 10.1371/journal.pone.0027104

**Published:** 2011-11-03

**Authors:** Caroline M. Hammerschlag-Peyer, Lauren A. Yeager, Márcio S. Araújo, Craig A. Layman

**Affiliations:** Department of Biological Sciences, Marine Sciences Program, Florida International University, North Miami, Florida, United States of America; National Institute of Water & Atmospheric Research, New Zealand

## Abstract

Ontogenetic niche shifts occur across diverse taxonomic groups, and can have critical implications for population dynamics, community structure, and ecosystem function. In this study, we provide a hypothesis-testing framework combining univariate and multivariate analyses to examine ontogenetic niche shifts using stable isotope ratios. This framework is based on three distinct ontogenetic niche shift scenarios, i.e., (1) no niche shift, (2) niche expansion/reduction, and (3) discrete niche shift between size classes. We developed criteria for identifying each scenario, as based on three important resource use characteristics, i.e., niche width, niche position, and niche overlap. We provide an empirical example for each ontogenetic niche shift scenario, illustrating differences in resource use characteristics among different organisms. The present framework provides a foundation for future studies on ontogenetic niche shifts, and also can be applied to examine resource variability among other population sub-groupings (e.g., by sex or phenotype).

## Introduction

Changes in resource use with body size or age, i.e., ontogenetic niche shifts, may occur in 80% of animal taxa [1], and have been shown to affect the structure and dynamics of populations, communities and ecosystems [1]–[3]. For instance, species often feed at higher trophic levels as they mature [4], [5] and thus, interactions with other species may shift from competition to predation through ontogeny [6], [7]. Many organisms increase their foraging range with ontogeny [8], thereby changing the nature of nutrient and energy flow through different habitats or ecosystems [9]. As such, ontogenetic niche shifts may render life stages as functionally distinct groups that should be considered as distinct nodes in food web models [10]. Hence, the study of ontogenetic niche shifts is of core interest in the ecological sciences.

**Figure 1:**
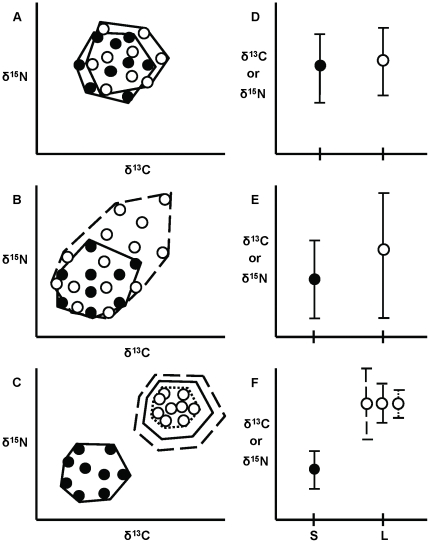
Representation of three possible ontogenetic niche shift scenarios using stable isotope ratios. Horizontally adjacent panels represent the same ontogenetic niche shift scenario. (A–C) Multivariate illustration of potential differences in niche width (represented by convex hull polygons), niche position and niche overlap (see text for more details) between two size classes in δ^13^C-δ^15^N niche space. (D–F) Univariate representation of niche width (variance of isotope values) and niche position (mean isotopic value) of either δ^13^C or δ^15^N between size classes. Closed circles represent isotope data of small individuals and open circles of large individuals. For B & E, this could also be a niche reduction, i.e., small individuals would occupy larger niche width than large individuals. Solid line  =  constant niche width, dotted line  =  niche reduction, dashed line  =  niche expansion; S  =  small size class, L  =  large size class. In panel F, solid line  =  constant variance, dotted line  =  reduced variance, dashed line  =  increased variance.

In a classic paper, Werner and Gilliam [11] proposed three possible scenarios for how an organism's resource use (e.g., diet, habitat use) may (or may not) change through ontogeny. First, a consumer may have no substantial ontogenetic changes in resource use (Fig. 1A, 1D). This scenario may occur in specialist taxa, such as phytophagus insects which are highly selective feeders throughout ontogeny [12]. Second, the niche of a smaller size class may be a subset of the niche of a larger size class, e.g., because larger individuals expand their foraging range and incorporate prey items that smaller individuals do not encounter (Fig. 1B, 1E; opposite scenario can also be true, i.e., niche of larger size class can be a subset of a smaller size class) [13]. Third, consumers may switch to a different resource pool during ontogenetic development (Fig. 1C, 1F), e.g., those organisms that have different diets following metamorphosis [14] or following shifts across habitat boundaries [15]–[17]. These different ontogenetic niche shift scenarios will translate into changes in niche width, niche position and/or niche overlap between size classes.

Stable isotope analysis often is applied to investigate ontogenetic niche shifts because it provides a time- and space-integrated representation of diet and/or is useful for those organisms whose diets are difficult to characterize directly [18], [19]. The majority of diet studies have employed stable isotope ratios of carbon (i.e., δ^13^C) and nitrogen (i.e., δ^15^N), as they provide information related to a consumer's basal resource pool and trophic position, respectively [19]–[21]. Most studies using stable isotopes to examine ontogenetic changes in diet rely on qualitative observations (i.e., drawing conclusions without using statistical descriptions or tests) or analyze δ^13^C and δ^15^N separately, either against a continuous body size gradient (e.g., regression analysis [22]–[24]) or among size/age groups (e.g., t-test, analysis of variance [25]–[27]; Fig. 1A–C). Yet, recent food web studies have shown the power of simultaneously analyzing δ^13^C and δ^15^N in order to better characterize overall patterns in niche variation [28]–[31]. For instance, bi- or multivariate analysis (e.g., simultaneous analysis of δ^13^C and δ^15^N) enables the detection of potential correlations between variables, which is not possible in univariate analysis [32].

Our aim was to provide a single hypothesis-testing framework that can delineate examinations of ontogenetic niche shift scenarios [11]. Our proposed framework incorporates both univariate and multivariate analyses to investigate shifts in niche width, niche position and niche overlap through ontogeny. We developed specific criteria characterizing each ontogenetic niche shift category and provide empirical examples to illustrate each. We hope this provides a unified framework for extending the classic niche shift categorization defined by Werner and Gilliam [11].

## Materials and Methods

We evaluated three niche aspects, including (1) niche width (variety in resources consumed), (2) niche position (types of resources consumed), and (3) niche overlap (similarity in resource use among individuals). We examined changes in niche width and niche position through ontogeny using multivariate and univariate analyses (see below). If niche width and/or niche position were found to differ through ontogeny using multivariate analysis, conventional univariate tests were performed to elucidate which niche axis (e.g., δ^13^C, δ^15^N) drove the observed niche shift (Fig. 1). For example, ontogenetic shifts in δ^13^C values could indicate dissimilar use of habitats or resource pools by different size classes [21], [33]–[35], whereas changes in δ^15^N values typically imply a shift in trophic position [21], [36], [37]. We then measured niche overlap between size classes in two-dimensional niche space (i.e., δ^13^C-δ^15^N-biplot space) using a % overlap measure [38]. Niche width, niche position and niche overlap are important aspects to identify ontogenetic niche shifts and can be used to classify an organism into one of the three categories proposed in the classic paper of Werner and Gilliam [11]. Following, we identify specific quantitative criteria that can be used for each of these niche shift scenarios.

**Figure 2:**
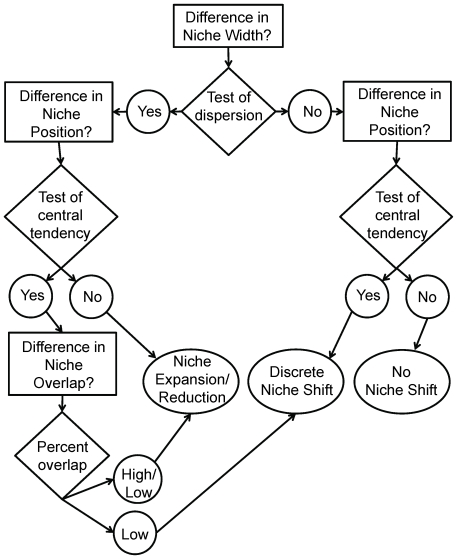
Flow chart of our method. Niche aspects (i.e., niche width, niche position, and niche overlap) are shown in rectangles, and test types in diamonds; “Yes”  =  niche width or niche position differs between size classes, “No”  =  niche width or niche position does not differ between size classes. The three possible scenarios are represented in ovals, with “No Niche Shift”  =  first scenario, “Niche Expansion/Reduction”  =  second scenario, and “Discrete Niche Shift”  =  third scenario. Low  =  low niche overlap for both size classes; High  =  high niche overlap for both size classes; High/Low  =  asymmetric niche overlap between two size classes.

The criteria for the first ontogenetic niche shift scenario, involving no change in diet through ontogeny are: no difference in (1) niche width and (2) niche position, imparting (3) a high degree of overlap in individuals' isotope values (Figs. 1A, 1D, 2). For the second scenario, breadth of resource use (i.e., diet or habitat use) is larger in one group than in the other, resulting in (1) a difference in niche width, irrespective of (2) niche position. More specifically, the isotopic niche width of one group is statistically larger than that of the other and the niche of the latter group is largely encompassed by the former, leading to (3) an asymmetry in niche overlap (Figs. 1B, 2). Niche position may or may not differ between groups, depending on whether resource expansion/reduction takes place from the center of the isotopic niche space (no niche shift) or is directed away from that center (Figs. 1B, 1E, 2). For the third scenario, involving a discrete ontogenetic diet shift, (1) niche width of one group can either be the same, smaller or larger than the other (Fig. 1C, 1F), with (2) a distinct shift in niche position, resulting in (3) little or no overlap in isotopic niche (Figs. 1C, 1F, 2).

We used empirical data to illustrate these different niche shift scenarios. We chose these examples specifically to illustrate the methodological framework discussed herein, and not as independent tests of the nature of niche shifts in these particular taxa. For these taxa, we collected direct diet data (or in one case, published diet information) to further help us characterize and understand niche variation. Post-metamorphic *Eupemphix nattereri* frogs (i.e., no tadpoles), gray snapper (*Lutjanus griseus*) and hardhead silversides (*Atherinomorus stipes*) constituted the model species.

Post-metamorphic *Eupemphix nattereri* specimens were collected from an area of Brazilian savannahs locally known as Cerrado in the municipality of Uberlândia (18°55′ S, 48°17′ W) in southeastern Brazil, a region characterized by shrubby grassland areas surrounding wet areas such as *veredas* (marshes with buriti-palms *Mauritia flexuosa*) or temporal and permanent ponds. Frogs were collected from October 1999 to October 2001 and immediately killed upon collection, preserved in 5% formalin and later transferred to 70% ethanol. Since all individuals were preserved in the same manner, differences in isotope values among individuals should have not been affected by preservatives [39]. Gut content analysis was performed via dissection and prey items were counted and identified to the lowest taxonomic level. Gut content data of *E. nattereri* are published elsewhere [39]. Upon dissection, individuals were sexed by examination of gonads and classified as adults if the gonads were fully developed (reproductive) or as juveniles if gonads were underdeveloped (non-reproductive). We used a piece of muscle from the thigh to measure δ^13^C and δ^15^N [39].

Gray snapper (*Lutjanus griseus*) were collected in the Loxahatchee River (26°57′ N, 80°06′ W) located on the southeast Atlantic coast of Florida, USA. Snappers were caught during the summers of 2007 – 2009 by angling and electrofishing in the mesohaline areas of the river. Fish were anesthetized using eugenol [40] and their standard length was measured. Each individual was forced to regurgitate their stomach contents by pressing on the abdomen while using a metal spatula to help invert the stomach [41]. Stomach content data of *L. griseus* are published elsewhere [41]. A small sample (∼1cm^2^) of dorsal fin tissue was taken from each fish for stable isotope analysis. After sampling their stomach contents, fish were returned to ambient water and allowed to recover before being released. Since the size range of *L. griseus* in the Loxahatchee River does not include reproductively mature adults, we *a priori* divided the individuals into juveniles (<100 mm SL) and sub-adults (≥100 mm SL) based on observed differences in habitat use between these two life-history stages [42], [43].

Hardhead silversides (*Atherinomorus stipes*) were collected by cast netting in a tidal creek (26°21′36.58″N, 77°00′58.91″W) on Abaco Island, Bahamas on November 15^th^ 2009. This creek is lined by mangroves (primarily red mangrove, *Rhizophora mangle*) and supports extensive seagrass beds predominantly consisting of turtle grass, *Thalassia testudinum*. The creek is dominated by marine waters with relatively little topographic relief, a small watershed, and little freshwater input [30]. All captured individuals were immediately put on ice and later frozen. The whole organism was used for stable isotope analysis. Diet information of *A. stipes* was obtained by Boveri and Quiros [44]. Based on gonad inspections, *A. stipes* was divided into juveniles (underdeveloped gonads) and adults (fully developed gonads).

We employed ratios of ^15^N to ^14^N and of ^13^C to ^12^C, and the stable isotope values are reported in the δ notation where δ^13^C  =  [(*R*_sample_ /*R*_standard_) – 1] ×1000, and where *R* is ^13^C / ^12^C and ^15^N / ^14^N, respectively. We focused on ratios of δ^15^N and δ^13^C because each reveals a distinct aspect of the consumer's long-term trophic niche (see above). PDB (PeeDee belemnite) and AIR (atmospheric nitrogen) are the global standards of δ^13^C and δ^15^N, respectively. Isotope sample preparation and analysis followed Post et al. [45] and was conducted at the Yale Earth System Center for Stable Isotopic Studies using a ThermoFinnigan DeltaPlus mass spectrometer (for *L. griseus* and *A. stipes*) and at the Centro de Energia Nuclear na Agricultura of the Universidade de São Paulo using a Micromass 602E mass spectrometer (for *E. nattereri*).

To evaluate for which ontogenetic niche shift scenario the model species met the criteria, we performed multivariate analyses, using δ^13^C and δ^15^N simultaneously, and “post-hoc” univariate analyses, separately on δ^13^C and δ^15^N. For the multivariate analyses, we first examined significant differences in (1) niche width and (2) niche position between the two groups, and then (3) niche overlap (Fig. 2). To do so, we performed a test for differences in dispersion and central tendency, respectively, following Turner et al. [46] in R version 2.12.2. In the context of this study, difference in dispersion represents a change in niche width because this metric measures the average trophic variability within size classes. More precisely, using analysis of nested linear models and residual permutation procedure, the mean distance to centroid (bivariate mean) was computed for each size class separately, and then the absolute value of the difference was evaluated between size classes. An absolute value greater than zero indicates a difference in niche width between size classes [46]. Similarly, the difference in central tendency represents a shift in isotopic niche position and was measured by computing the Euclidean distance between the centroids of the two groups [46]. The isotopic niche position was considered to be different if the Euclidean distance between the two groups was significantly greater than zero (R codes for the test of dispersion and central tendency are provided in the Appendix of Turner et al. [46]). The test statistics for dispersion and central tendency are herein referred to as “mean distance to centroid” and “Euclidean distance”, respectively.

Conventional univariate analysis was applied after significant results from multivariate analysis to provide additional detail. To this end, we first tested all data for normality (Shapiro-Wilk test) and square-root transformed them when applicable. Then, we examined shifts in niche width and niche position for each stable isotope element by measuring (1) homogeneity of variance between size classes using Bartlett's test and (2) by comparing mean isotopic values between size classes using t-test for independent samples (for normally distributed data) or Wilcox test (for non-normally distributed data). All tests were performed in R version 2.12.2. Significance was declared at α≤0.05.

**Figure 3:**
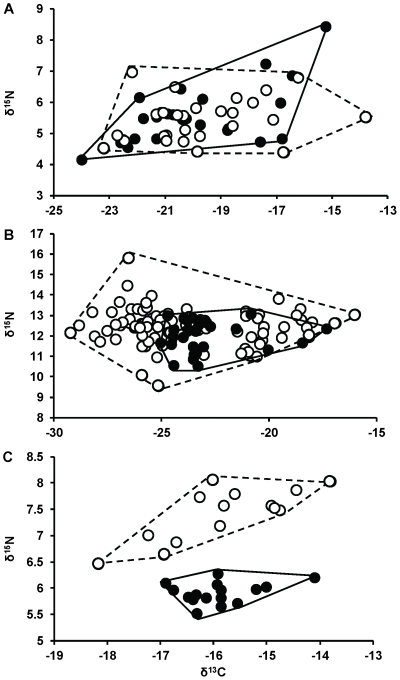
Isotopic niche use of two ontogenetically distinct groups. Differences in niche width (for illustration purposes represented by convex hull polygons) of a small (solid line) and large (dashed line) size class of A) *Eupemphix nattereri,* B) *Lutjanus griseus,* and C) *Atherinomorus stipes* represented in a δ^13^C-δ^15^N niche space. Closed circles represent isotope data of individual juveniles and open circles individual adults (or sub-adults in case of *L. griseus*).

We measured niche overlap between groups by quantifying, for each group, the percentage of individuals that were encompassed by the other group's convex hull [38], which is the area of the smallest convex polygon that contains all individuals of a group in a δ^13^C-δ^15^N-biplot (Figs. 1, 3) [47].The convex hull approach offers some advantages for characterizing niche width when compared to alternative analyses. The convex hull approach is powerful because it incorporates each individual of the population's sub-sample, and thus includes information about the niche width of the population including every sampled individual. Conversely, other approaches are targeted at identifying the “core” niche of the population, a niche metric which could exclude particular individual niches from the characterization of the population niche [28]. Either of these approaches may be more relevant with respect to a particular question of interest and/or the nature of the underlying data set. Herein, we chose to measure niche overlap based on the convex hull approach, as the importance of individual level niche variation is increasingly recognized as an important component of ecological dynamics and evolutionary trajectories [48], [49].

Since for many organisms body size is more important in determining life history characteristics than age per se [50], we used body size as a proxy for ontogenetic stage. More specifically, we used categorical size classes instead of continuous body size data (Fig. 1). Yet, for the univariate analyses, our framework could easily be applied to a continuous body size gradient, e.g., using linear regression (to test for non-zero slope, instead of comparing means between groups) when examining shifts in niche position. When categorical size groups are used, biologically meaningful size classes should be chosen (as in this study), or a break point analysis [51] could be performed, to determine the size at which change in resource use occur.

In this paper we use a traditional, frequentist approach (i.e., null hypothesis significance testing based on *P*-values) to evaluate which ontogenetic niche shift models best represent our empirical examples. Yet, our analytical framework can easily be applied to alternative statistical approaches (e.g., information theory or likelihood ratios) [52]–[55] to select which model (i.e., ontogenetic niche shift scenario) fits best the empirical data used.

## Results

For the illustration of the first ontogenetic niche shift scenario, we used 25 post-metamorphic juveniles (size range: 13–33mm Snout-Vent Length (SVL)) and 26 adults (size range: 34–47mm SVL) of *E. nattereri.* Juvenile and adult *E. nattereri* did not statistically differ in their (1) niche widths (mean distance to centroid  = 0.08, *P* = 0.87), and (2) isotopic niche position (Euclidean distance  = 0.41, *P* = 0.58; Fig. 3A). Individuals of both groups (3) overlapped substantially with each other (juveniles  = 92% overlap with adults, adults  = 85% overlap with juveniles, Fig. 3A).

Juvenile (n = 31, size range: 40–96mm Standard Length (SL)) and sub-adult (n = 89, size range: 101–204mm SL) *L. griseus* differed significantly in their (1) niche width (mean distance to centroid  = 1.22, *P* = 0.006, Fig. 3B), which was driven by a difference in variance of δ^13^C values (Bartlett: K^2^ = 10.37, df = 1, *P* = 0.001), not δ^15^N (Bartlett: K^2^ = 1.07, df = 1, *P* = 0.3). There was no shift in (2) isotopic niche position (Euclidean distance  = 0.94, *P* = 0.13; Fig. 3B) and (3) most juvenile *L. griseus* overlapped with the niche width of sub-adults (97% of individuals), whereas only 35% of sub-adults were encompassed by the convex hull of the juveniles (Fig. 3B).

Juveniles (n = 16, size range: 23–35mm SL) and adults (n = 14, size range: 40–61mm SL) of *A. stipes* differed significantly in (1) niche width (mean distance to centroid  = 0.51, *P* = 0.01; Fig. 3C), which was caused by differences in variance of δ^15^N (Bartlett: K^2^ = 10.6, df = 1, *P* = 0.001), as well as δ^13^C (Bartlett: K^2^ = 3.85, df = 1, *P* = 0.05). In addition, the (2) isotopic niche position changed significantly between juvenile and adult *A. stipes* (Euclidean distance  = 1.5, *P*<0.0001; Fig. 3C), which was driven by a change in their mean δ^15^N values (Wilcox: W = 224, *P*<0.001), but not mean δ^13^C values (t-test: t = 0.29, df = 20.3, *P* = 0.77). (3) No individuals were encompassed by the convex hull of the other group (Fig. 3C).

## Discussion

Because of the significant effects ontogenetic niche shifts can have on the structure and dynamics of populations, communities and ecosystems, it is important to identify the nature of these dietary shifts using quantitative techniques [1]–[3]. Stable isotope analysis is especially useful for this purpose because of its time- and space-integrated representation of diet [47], [56], [57]. Yet, most studies using stable isotope ratios have examined ontogenetic niche shifts either qualitatively or by analyzing stable isotope elements separately [23], [58]–[61]. Quantitative measures analyzing isotope elements simultaneously are advantageous in identifying the nature of dietary shifts through ontogeny, offering increased knowledge of potential shifts in niche width, niche position and niche overlap, and can detect possible correlations among these elements [32]. This study provides a hypothesis-testing framework to investigate ontogenetic niche shifts in organisms by applying univariate and multivariate analyses simultaneously on stable isotope elements. In doing so, we provide a foundation for exploring ontogenetic niche shifts in any organism of interest.

Post-metamorphic juveniles and adults of *E. nattereri* illustrate the first ontogenetic niche shift scenario: there were no differences in niche width and niche position between the two size classes, and they overlapped greatly (Fig. 3A). Since frogs can grow substantially after metamorphosis, they could be expected to experience considerable diet shifts during the terrestrial phase of their life cycle [11], but this was not found to be the case. Stomach content analysis supported the isotope analysis findings by showing that both juvenile and adult *E. nattereri* tend to specialize on ants and termites [39]. Since stable isotope ratios of muscle tissue represent diet over a long time period (weeks to months, [62]), it can be inferred that the observed diet specialization was long-term, and not just based on local prey availability at the time of sampling (an advantage of stable isotope analysis over stomach content analysis, [63]).

Gray snapper illustrate the second ontogenetic niche shift scenario: sub-adult *L. griseus* expanded their isotopic niche to include diet items with more depleted δ^13^C values (Fig. 3B). Direct diet analysis confirmed that the feeding of juvenile *L. griseus* was essentially confined to the oyster reef matrix of the Loxahatchee River, where their diet was composed almost entirely of oyster reef-associated prey items (i.e., mud crabs, *Eurypanopeus* sp. and *Panopeus* sp.). Conversely, sub-adult *L. griseus* move to the adjacent mangrove habitats to feed on mangrove-associated prey (i.e., green mangrove tree crab, *Aratus pisonii*) [41]. Prey items in oyster reef habitats are largely supported by microalgae- and phytoplankton-based trophic pathways that are more enriched in δ^13^C values (∼ -18 ^0^/_00_), whereas prey from mangrove-based food web modules are more depleted (δ^13^C ∼ -27 ^0^/_00_) [41], [64]. Sub-adults most likely increased their foraging area because of decreased predation pressure or increased mobility due to larger body size [1]. Such foraging and predation risk trade-offs and/or increase in mobility with body size can drive many ontogenetic niche shifts, and stable isotope ratios can be a prime tool to reflect such long-term feeding shifts when isotopic signatures of sources are distinct.

Juvenile and adult *A. stipes* displayed a distinct niche shift, mainly along the δ^15^N axis (Fig. 3C). Since *A. stipes* is a visual feeder that actively selects zooplankton [44], no major ontogenetic niche shift would be expected for that species. Yet, our stable isotope data suggest that adults likely fed exclusively on larger-sized zooplankton prey, as larger zooplankton are often more enriched in δ^15^N [24]. Since adult and juvenile *A. stipes* share the same resources (i.e., habitat and diet), adults might shift to larger prey sizes as a means to reduce intrapopulation niche competition [49].

Our empirical examples highlight the benefit of using both univariate and multivariate measures, as each was useful to identify different aspects of the niche differences. For example, in the case of gray snapper, multivariate approaches were useful in identifying degree of niche width and niche overlap, whereas univariate analysis was important to elucidate niche expansion in the larger size class primarily along the carbon axis. It would be difficult to differentiate among the three major niche shift scenarios by using univariate analyses alone (Fig. 1D–F).

When applying the proposed framework, it is important to consider that the three ontogenetic niche shift scenarios outlined in this study should be understood as endpoints of a continuum. Many organisms might fall between the endpoint scenarios. In addition, statistical significance does not always equate to biological importance, and vice versa [65], and thus caution should be exerted when interpreting empirical data. The much discussed limitations of isotopes must also be considered when interpreting their application to study ontogenetic diet shifts [19], [20], [66]. For instance, source pools need to have distinct isotopic signatures for stable isotopes to be useful, and δ values can be particularly sensitive to spatial and temporal variation in isotope values of source pools. As such, scattering among consumers in a δ^13^C-δ^15^N biplot could be the result of a broad resource use among individuals, or due to high variation in isotope values of source pools. Consequently, the use of a complimentary method such as stomach content analysis (as applied in this study), fecal analysis, or direct observations are useful to interpret and better understand patterns in isotope signatures. When stable isotope ratios are put in the proper context, they can be a very powerful tool [66] and provide insights that would not be possible with some conventional methods [19].

Intrapopulation resource variation has critical ecological, evolutionary and conservation implications [48], [49], and ontogenetic niche shifts are one primary driver of this variation [48]. Our approach provides a framework for exploring questions related to ontogenetic diet shifts, as well as other among-group (e.g., sex or phenotype) comparisons. Such studies are critical for understanding interactions among individuals at population, community and ecosystem levels.
